# Investigating Parietal and Premotor Influence on Motor Cortical Excitability Associated with Visuomotor Associative Plasticity

**DOI:** 10.3390/brainsci11040452

**Published:** 2021-04-02

**Authors:** Paul J. Wolfe, Lynea B. Kaethler, W. Richard Staines

**Affiliations:** Department of Kinesiology, University of Waterloo, 200 University Ave. Waterloo, ON N2L 3G1, Canada; pjwolfe@uwaterloo.ca (P.J.W.); lkaethler@uwaterloo.ca (L.B.K.)

**Keywords:** visual paired associative stimulation, V-PAS, motor cortex, parietal, premotor, SPOC, PMd, adaptive plasticity

## Abstract

The brain changes in response to sensory signals it is exposed to. It has been shown that long term potentiation-like neuroplasticity can be experimentally induced via visual paired-associative stimulation (V-PAS). V-PAS combines afferent visual stimuli with a transcranial magnetic stimulation pulse to induce plasticity. Preparation of a reaching movement to generate activity in superior parietal occipital cortex (SPOC) was used in this study as an additional afferent contributor to modulate the resultant plasticity. We hypothesized that V-PAS with a reaching movement would induce greater cortical excitability than V-PAS alone and would exhibit facilitated SPOC to M1 projections. All four experiments enrolled groups of 10 participants to complete variations of V-PAS in a repeated measures design. SPOC to M1 projections facilitated motor cortex excitability following V-PAS regardless of intervention received. We did not observe evidence indicating extra afferent information provided an additive effect to participants. Investigation of PMd to M1 projections confirmed disinhibition and suggested interneuronal populations within M1 may be mechanistically involved. Future research should look to rule out the existence of an upper limit for effective afference during V-PAS and investigate the average influence of V-PAS on cortical excitability in the larger population.

## 1. Introduction

A hallmark of the human brain is the ability to continuously adapt and learn from the environment through neuroplasticity. It is becoming clear long-term potentiation (LTP) is critically involved in this process. Paired-associative stimulation (PAS) is one of several approaches documented in the literature which can create conditions in the brain similar to LTP. PAS functions with the principle of Hebbian plasticity which formalizes the observation when one neuron repeatedly stimulates another neuron, the synapse between those neurons will change to strengthen that connection [1]. This mechanism is manufactured in PAS which pairs a sensory stimulus with a single pulse of transcranial magnetic stimulation (TMS) repeated over many trials. The events are timed such that the sensory stimulus arrives to the site of TMS in the primary motor cortex (M1) just as TMS is delivered to simulate the natural firing of the involved neurons. Correct timing between stimuli (interstimulus interval (ISI)) is imperative in this paradigm. Previous research has shown that LTP-like plasticity is observed when the sensory stimulus arrives just prior to TMS onset; however, when TMS onset occurs before the sensory stimulus can arrive in M1, an opposite effect is induced [2]. The ISI interval required is dependent on the site of the peripheral stimulus. For example, a common application of PAS stimulates the median nerve followed by a single TMS pulse 25 ms later [3,4,5,6].

While the ability to stimulate various peripheral nerves provides a degree of versatility to PAS, the traditional protocol restricts stimulation to the somatosensory domain. Recently, this boundary has been extended to include the visual domain [7]. Termed V-PAS, this protocol pairs the M1 TMS pulse with a visual stimulus rather than a peripheral nerve stimulation. V-PAS relies on the same principle as traditional PAS of combining a sensory signal with TMS over M1. Two major differences arise however: first, the required ISI is of longer duration due to the relatively slow visual system processing time; second, many more repetitions are required to observe a lasting effect [7].

Early investigation of V-PAS suggests the dorsal premotor cortex (PMd) is involved in facilitating LTP-like plasticity following V-PAS [7]. The PMd is associated with the preparation and selection of motor execution for a given task [8,9,10,11] and receives substantial input from anterior and posterior parietal cortex (PPC) which in turn are connected with regions of the visual cortex [12,13]. Of the various regions within the PPC, the superior parietal occipital cortex (SPOC) is one such parietal region which projects to PMd [14,15,16]; we targeted SPOC in this study due to these projections. SPOC is observed to activate specifically when a reaching task is required [17,18,19,20], independent from the end goal of the motion (such as grasping an object) [21].

The convergence of various sensorimotor networks in PMd was exploited in the present study. Given that all variations of PAS share the commonality of pairing a sensory stimulus with TMS, we designed the present study to investigate if plasticity induction is influenced by incorporating additional, time-locked cortical activity by evaluating M1 excitability following V-PAS alone and V-PAS in combination with a visually guided reach. In separate experiments the contributions of the SPOC (Experiments 1 and 2) and the PMd (Experiments 3 and 4) to these V-PAS induced M1 adaptations were evaluated with paired-pulse TMS. We hypothesized that the increased activity directed to PMd will facilitate a greater degree of neuroplasticity induction compared to V-PAS without a reaching component. It was further hypothesized that there would be contributions from functionally connected premotor and parietal areas to M1 excitability adaptations, specifically that there would be a disinhibition of PMd-M1 and a facilitation of SPOC-M1 interactions following V-PAS coupled with motor training.

## 2. Materials and Methods

### 2.1. Participants

Thirty healthy right-handed young adults (between 19–30 yrs; Mean 22.2 yrs; 13 males; 17 females) participated in this study after providing written informed consent. All participants were free of neurologic illness and passed visual acuity and stereoacuity testing prior to enrollment. Visual acuity was assessed via Snellen chart where participants all achieved at least 20/20 in both monocular and binocular viewing conditions. To demonstrate adequate stereoacuity, participants were required to achieve ≥20″ on the Randot Stereo test. All participants completed a TMS screening form [22] to ensure there were no contraindications to TMS. All experimental procedures received ethics clearance from the Office of Research ethics at the University of Waterloo and conformed to the Declaration of Helsinki.

### 2.2. Localization of Brain Sites

To aid TMS navigation, we used a Brainsight neuronavigation system (Brainsight; Rogue Research, Montreal, QC, Canada). The hand representation was defined as the location within left M1 that evoked the greatest muscle activity in the right first dorsal interosseous (FDI) muscle of the hand when probed via TMS. The TMS coil was positioned 45° to the midline, tangential to the scalp. SPOC was localized through Brainsight (Talairach coordinates: x = −9, y = −74, z = 41). These coordinates are mean values determined experimentally through previous fMRI research [18,23,24,25] and are comparable to previous studies [17,21] thereby lending confidence to our methods in lieu of individual fMRI scans in the present study. The coil was held over SPOC tangential to the scalp along a parasagittal line rotated approximately 15°. These coil positions were organized to induce posterior-anterior current flow while also creating space for both coils to be positioned simultaneously. In addition, the left PMd was located 2.5 cm anterior and 1 cm medial to the M1_FDI_ location [26]. Thresholding was repeated for the CS coil over the M1_FDI_ location, in the position it would be in to stimulate the PMd, which partially overlaps the TS coil. This is necessary because the stimulus intensity rapidly dissipates as the distance between the coil and the surface of the skull increases.

### 2.3. Outcome Measures

All outcome measures were completed using a biphasic Magstim TMS stimulator (Model: Magstim^2^, Magstim, Whitland, UK). Connected to the stimulator were two figure-of-eight coils with the capacity to activate each coil independently or coordinated together. Once the FDI motor hotspot was located, resting motor threshold (RMT) was determined to ensure consistent stimulation across participants. We defined RMT as the lowest stimulator intensity required to evoke an MEP of at least 50 µV in 5 of 10 consecutive trials.

Surface bipolar Ag/AgCl electrodes were placed over the right FDI to record MEPs. Raw EMG was amplified 1000× and band-pass filtered between 2 Hz and 2.5 kHz then digitized at 5 kHz and recorded using Signal software (Cambridge Electronic Devices, Cambridge, UK) for later analysis.

### 2.4. Single-Pulse TMS

A single TMS coil was positioned over the motor hotspot which delivered individual biphasic pulses to participants. Intensity was selected to reliably evoke a 1 mV MEP during pre-testing, this intensity was recorded and used during post-testing. Twenty-four individual MEPs were recorded and observed; any trials which did not produce a clear MEP were immediately discarded and recollected.

### 2.5. Paired-Pulse TMS

Local intracortical interactions were assessed using one TMS coil over three configurations. Each configuration applied two pulses of TMS over the FDI motor hotspot with set ISIs between them. Resultant paired-pulse MEPs were compared to single pulse control MEPs to quantify how each configuration altered efferent activity. Short-interval intracortical inhibition (SICI) was observed by applying a CS of 80% RMT followed by a 120% RMT TS 2.5 ms later. We evoked intracortical facilitation (ICF) using an 80% RMT CS paired with a 120% RMT TS 10 ms after. Lastly, long-interval intracortical inhibition (LICI) was observed by using a 120% RMT CS and a 120% RMT TS separated by 100 ms.

**Figure 1:**
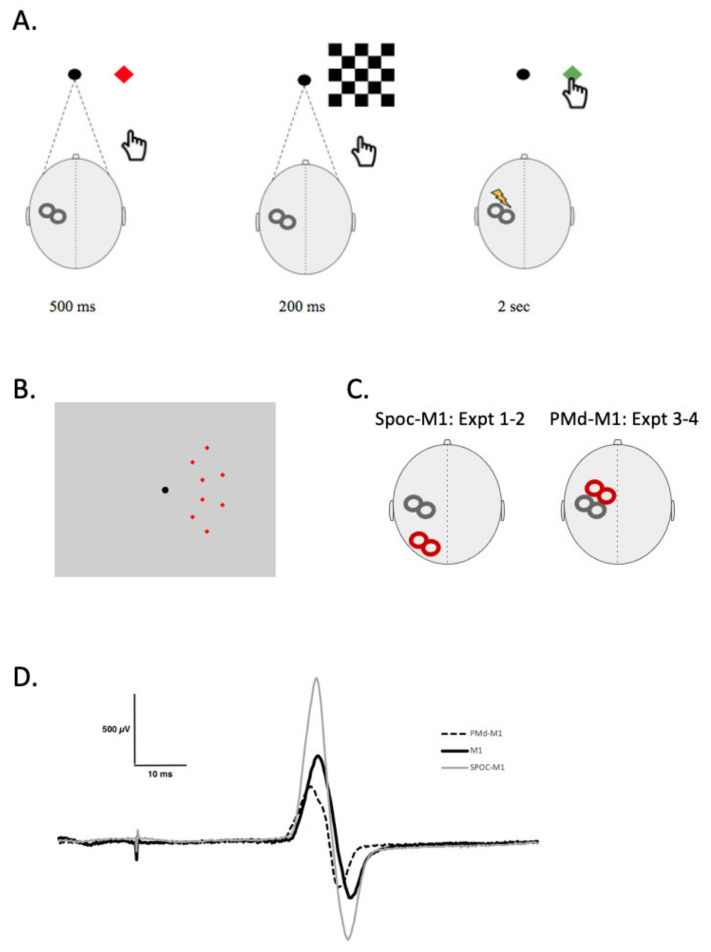
V-PAS + motor training task. (**A**) shows a single V-PAS + motor training trial. The far left shows the target location appearance in red for 500 ms, immediately followed by the visual stimulus appearance for 200 ms. At the disappearance of the visual stimulus the TMS stimulus is delivered, simultaneously with the target reappearance in green; the cue to move. For V-PAS the reach action was omitted and for the motor training intervention, the TMS stimulus was omitted. The participant has 2 s to reach and touch the target before the next trial begins. (**B**) Image shows all eight possible target locations on the computer screen. (**C**) Schematic representation of TMS coil positions during Experiments 1 and 2 (SPOC-Talairach coordinates: x = −9, y = −74, z = 41) and Experiments 3 and 4 (PMd—2.5 cm anterior and 1 cm medial to M1_FDI_ location). (**D**) Example MEP traces following a: single TMS pulse (black), conditioning pulse over SPOC (gray) 4 ms prior, and conditioning pulse over PMd (dashed) 6 ms prior.

Paired-pulse TMS (ppTMS) was also included to test the projections from SPOC to M1 or from PMd to M1 using two TMS coils (Figure 1C). The conditioning stimulus (CS) was delivered over SPOC or PMd at an intensity of 90% resting motor threshold (RMT) followed by the test stimulus (TS) of 120% RMT over the FDI motor hotspot 4 ms (SPOC) [21] or 6 ms (PMd) [7] later. Sample MEPs from an individual participant are shown for these 2 conditions in Figure 1D. Participants experienced this procedure in two conditions: while at rest and while actively performing movement training. Twenty-four MEPs were collected for each condition. During this measure, participants viewed (and interacted with during movement training) the same apparatus used during V-PAS, except with the removal of the checkerboard presentation (see V-PAS below).

### 2.6. Motor Training Task

Participants were seated comfortably in front of a 19″ monitor (1280 × 1024 resolution) with their right arm resting outstretched in front of them for the duration of the study. This screen presented participants a solid black central fixation circle (40 cm from participant, 0.72°) in addition to components used in paired-pulse TMS and V-PAS. During training, participants interacted with the visual stimuli by reaching to touch the screen with the tip of the right index finger. Motor training involved repeated trials of target presentation met with a reach-to-touch motor response. Trials began by presenting a solid red square cue target (1.15°) in one of eight possible locations on the right side of the screen. Targets were positioned along two arcs spaced 9.29° and 14.25° away from the fixation point with four targets evenly spaced in each arc (Figure 1B) both above and below centre. The cue informs participants where they will need to touch on the screen when the signal to begin appears 500 ms later. Following this preparatory phase, the red square turns to green at which time the participant is instructed to touch the target as quickly but safely as possible. Participants have two seconds to execute the movement and return to the starting position before the next trial begins. Procedures in this study were organized into repeated blocks of 8 trials to allow random presentation order of targets while ensuring that each location was presented an equal number of times.

### 2.7. V-PAS

A Magventure stimulator (Model: MagPro R30, Magventure, Alpharetta, GA, USA) attached to a figure-eight butterfly coil (MCF-B65) was used for the V-PAS intervention within each session. The intervention phase saw two additions to the motor training protocol. First, a checkerboard pattern was displayed on the right side of the screen between the red and green square targets for 200 ms. The pattern was nine checks tall by five wide, each check subtended 5.44° vertically and 4.58° horizontally and alternated solid black and white. Each trial also saw the introduction of a single pulse of TMS timed 200 ms following the onset of the checkerboard stimulus. TMS was localized over the FDI motor hotspot and was set to 120% RMT. In this way, one trial of V-PAS consisted of a cue presented for 500 ms followed by a checkerboard stimulus for 200 ms, culminated by a single pulse of TMS and the presentation of the green target for 2 s (Figure 1A). Participants engaged with V-PAS while at rest or while performing motor training (see experiment overviews below). Each session of V-PAS consisted of 304 trials delivered over approximately 15 min which corresponded to a TMS stimulus frequency of 0.37 Hz.

During presentation of visual stimuli in both the motor training and V-PAS procedures participants rested their chin in a chin rest and were instructed to maintain fixation on a black circle presented in the centre of the visual display (Figure 1B). Additionally, in experiments 1 and 2 electrooculographic (EOG) recordings were made to confirm that there were no eye movements during VPAS.

### 2.8. Study Design

This study included four experiments. Experiments 1 and 2 enrolled the same sample (*n* = 10), an average of seven days elapsed between these two sessions. Experiments 3 and 4 enrolled a new sample (*n* = 20) who completed one or both experiments.

#### 2.8.1. Experiment 1

The first experiment aimed to observe any changes with SPOC to M1 projections related to V-PAS or V-PAS combined with motor training. All participants first completed a session of V-PAS alone. During this session, all visual cues used for motor training were provided, however participants were instructed to maintain centre fixation on the fixation target and ignore the other cues. Participants returned approximately seven days later for a V-PAS + motor training session. This session now instructed participants to interact with the visual cues presented to them. Ultimately these cues guided participants to naturally plan and execute a reach-to-touch movement coordinated with a single pulse of TMS. Outcome measures were limited to paired-pulse TMS between SPOC and M1 during this experiment. Data were collected prior to and following the V-PAS interventions in both a resting and active condition and amplitudes were compared to single pulse MEPs alone.

#### 2.8.2. Experiment 2

Experiment 2 was conducted simultaneously with experiment 1 and shared the participant sample. This experiment investigated the influence of V-PAS and V-PAS + motor training on motor cortical excitability using single pulse MEPs of the FDI and on receptor changes as assessed by SICI, ICF, and LICI. These measures were observed both prior to and following each of the V-PAS interventions.

#### 2.8.3. Experiment 3

Experiment 3 was similar in design to Experiment 1, but it aimed to observe any changes with PMd to M1 projections related to V-PAS or V-PAS combined with motor training. Outcome measures were limited to paired-pulse TMS between PMd and M1 during this experiment. Data were collected prior to and following the V-PAS interventions in both a resting and active condition and amplitudes were compared to single pulse MEPs alone. Following the training, participants rested for ten minutes since no significant changes in excitability have been observed until ten minutes (and up to 30 min) after training [7], before the postintervention measures were collected. Two groups of 10 participants were included in Experiment 3 and for those who participated (*n* = 3) in both the V-PAS and V-PAS combined with motor training, the sessions were separated by at least 1 week.

#### 2.8.4. Experiment 4

Experiment 4 investigated the effect of motor training alone on PMd to M1 projections for comparison to Experiment 3. Ten participants (7 also participated in Experiment 3) performed the motor training task alone without the inclusion of V-PAS. Outcome measures were limited to paired-pulse TMS between PMd and M1 during this experiment. Data were collected prior to and following motor training alone, with a 10 min rest period immediately following training. Following the 10 min rest period, the single pulse MEPs and the rest and active paired pulse MEPs were collected, to assess the excitability changes in M1 and in the inhibitory connection between PMd and M1. The post-V-PAS training MEP collections followed the same stipulations as the pre-training MEP collections.

### 2.9. Data Analysis

MEPs were measured as peak-to-peak values. To exclude anticipatory responses (i.e., in which the motor output coincided with the TMS pulse), trials in which any EMG activity was present during the movement preparation period were removed (<1% of trials). Within participants, twenty-four trials were collected for averaging in each condition and paired pulse data was normalized to each participant’s corresponding average single pulse MEP value. For single pulse MEP, SICI, ICF, and LICI data separate 2 × 2 repeated measures ANOVAs with factors of Time (Pre/Post) within session and V-PAS (No Training/Training) between session were conducted. SPOC-M1 paired-pulse TMS data were first tested using a 3-way repeated measures ANOVA with factors of Time (Pre/Post), V-PAS (No Training/Training), and Activity (Rest/Active). This was followed with separate 2-way repeated measures ANOVAs across the V-PAS condition (No Training/Training) such that each ANOVA had factors of Time (Pre/Post) and Activity (Rest/Active). Similarly, PMd-M1 paired-pulse data in Experiments 3 and 4 were tested with 2-way repeated measures ANOVAs across the V-PAS sessions (No Training/Training, Expt 3) or the motor training session (Expt 4) such that each ANOVA had factors of Time (Pre/Post) and Activity (Rest/Active). Planned contrasts were used to test the hypothesis that V-PAS with Training will facilitate a greater degree of neuroplasticity induction (i.e., reduce PMd-M1 inhibition) in the active state. Data sets were assessed for normality and homogeneity of variance to ensure that the assumptions for performing the ANOVA were upheld. Statistical analysis was conducted with SAS and significance was taken as *p* < 0.05.

## 3. Results

### 3.1. Experiment 1

**Figure 2:**
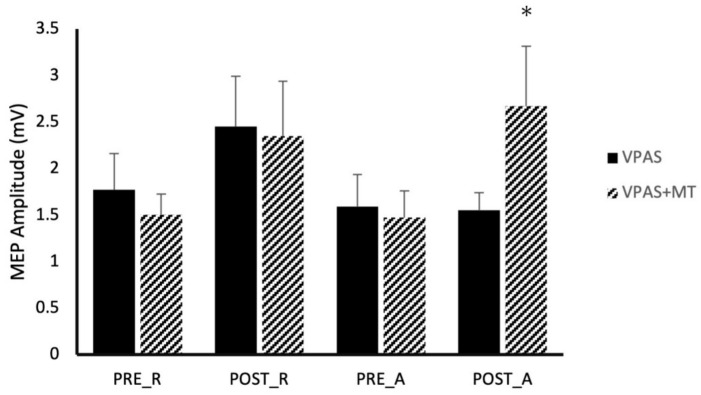
Effect of V-PAS and V-PAS + motor training on MEP amplitudes evoked by single-pulses to M1 and paired-pulses to SPOC and M1. Histograms represent mean paired-pulse MEP amplitudes which have been normalized to rest state single-pulse MEP amplitudes, showing the SPOC influence on M1 excitability at rest and during action preparation. Given the facilitatory effect of SPOC -M1 interactions, all normalized MEP amplitudes are greater than 100% of the single pulse MEP amplitudes. This graph shows significant increases in normalized MEP amplitudes after V-PAS + motor training which were not present in V-PAS alone in either the active or resting state. Asterix denotes significance (*p* < 0.05).

**Figure 3:**
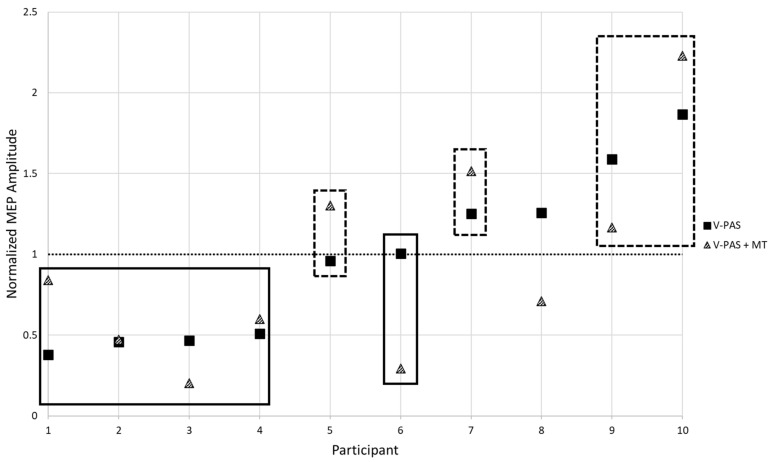
Participant responses to V-PAS (Solid Squares) and V-PAS + motor training (Dashed Triangles). MEP amplitude is normalized to baseline before V-PAS. Evident is the variable influence on excitability changes following V-PAS. Highlighted are groupings applied to the sample based upon average participant response to V-PAS. Participant 8 did not exhibit a consistent response and was excluded from a grouping.

SPOC-M1 paired-pulse TMS was examined over eight different conditions generated by combinations of: time (Pre/Post intervention), activity (Active/Rest), and V-PAS (No Training/Training) (Figure 2). Across all conditions, paired-pulse TMS resulted in significantly larger MEP size compared to MEPs evoked by a single pulse of TMS at the same stimulator intensity (*p* < 0.05). A 3-way, repeated measures ANOVA with factors time, activity, and V-PAS, revealed a significant 3-way interaction in the data (F_1,9_ = 8.82, *p* < 0.021) in addition to a main effect of time (F_1,9_ = 6.47, *p* = 0.032). We followed-up with two, 2-way repeated measure ANOVAs using time and activity as factors. Within the V-PAS “No Training” data, we found no significant effect of time (F_1,8_ = 1.15, *p* = 0.315) nor activity (F_1,8_ = 2.59, *p* = 0.146) and did not reveal a significant interaction effect (F_1,8_ = 1.06, *p* = 0.333). Lastly, within the V-PAS “Training” data, a main effect of time was revealed (F_1,9_ = 8.55, *p* = 0.017) while there was no significant main effect of activity (F_1,9_ = 0.01, *p* = 0.930) or interaction (F_1,9_ = 1.69, *p* = 0.226). Figure 3 depicts the effect of each V-PAS intervention across individual subjects. MEP amplitudes are normalized to baseline before V-PAS.

### 3.2. Experiment 2

We examined single pulse MEP data through a two-way repeated measures ANOVA with factors time (Pre/Post intervention) and V-PAS (No Training/Training). At each collection point, twenty-four MEPs were recorded while the participant remained at rest. Results showed no main effect of time (F_1,9_ = 0.25, *p* = 0.632) nor a significant interaction (F_1,9_ = 0.00, *p* = 0.948) in the data; however, a significant main effect of V-PAS was observed suggesting MEP sizes were significantly reduced throughout the training V-PAS session compared to the no-training V-PAS session (F_1,9_ = 14.83, *p* = 0.004). However, this effect of V-PAS session appears to be driven by differences in MEP amplitudes at baseline between sessions (Pre-V-PAS No Training/Training: 1.17 ± 0.13 mV/0.97 ± 0.13 mV; *p* < 0.05).

For each of SICI, ICF, and LICI, ten trials were averaged together before and after each V-PAS intervention. Each outcome measure was analyzed using a two-way repeated measures ANOVA with factors of time and V-PAS. In general, SICI, ICF, and LICI all presented a high degree of intersubject variability. Across all measures, we did not observe any significant main effects or interactions (*p* > 0.05).

### 3.3. Experiment 3

Pre-intervention measures from both V-PAS (No Training/Training) groups were analyzed to assess the state differences and influence of the PMd on M1. A comparison of the PMd-M1 paired-pulse to the single-pulse data confirmed an inhibitory effect of the PMd on M1 (*p* = 0.005). There was a significant effect of state, where Active showed significantly greater amplitude than Rest, in both single-pulse MEP amplitudes (*p* = 0.020) and paired-pulse MEP amplitudes (*p* = 0.014). Paired-pulse MEP amplitudes normalized to the respective state single pulse MEP amplitudes showed no significant effect of state.

**Figure 4:**
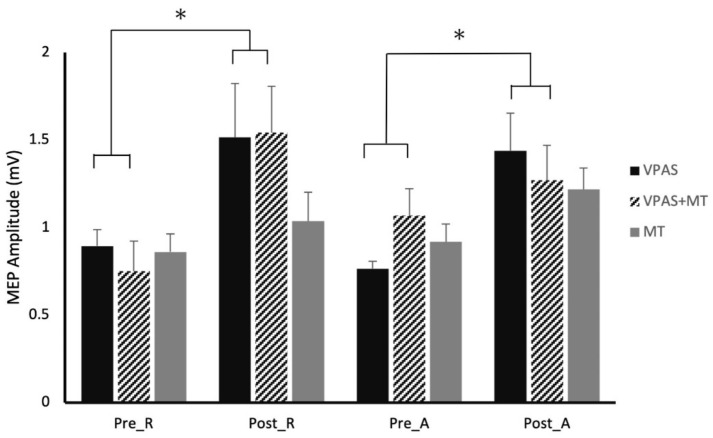
Effect of V-PAS, V-PAS + motor training, and motor training alone on MEP amplitudes evoked by single-pulse to M1 and paired-pulses to PMd and M1 (Experiments 3 and 4). Histograms represent the mean paired-pulse MEP amplitudes which have been normalized to the respective state single-pulse MEP amplitudes, showing the PMd influence on M1 excitability at rest and during action preparation. Sub-1 values are representative of the inhibitory influence the PMd exerts over M1 while the averages greater than one are indicative of the release of inhibition and facilitation of the PMd influence over M1. The graph shows the significant increases in MEP amplitude in both rest and active states following V-PAS, as well as significant increases in rest state MEP amplitude following V-PAS + motor training. Significant increases in overall MEP amplitude were seen after the motor training intervention. Asterix denotes significance (*p* < 0.05).

Within the V-PAS “No Training” data, there were no main effects or interactions for single-pulse MEPs (*p* > 0.05). For PMd-M1 paired-pulse data, there was a main effect of time (F_1,27_ = 10.91, *p* = 0.003) but not of activity (F_1,27_ = 0.25, *p* = 0.622), nor was there a significant interaction (F_1,27_ = 0.03, *p* = 0.866). The contrasts confirmed that the main effect of Time was driven by both changes in the rest and active state data (Rest: F_1,27_ = 4.90, *p* = 0.035; Active: F_1,27_ = 6.03, *p* = 0.021) (Figure 4).

Within the V-PAS “Training” data, there were no main effects or interactions for single-pulse MEPs (*p* > 0.05). For PMd-M1 paired-pulse data, there was a main effect of time (F_1,27_ = 4.98, *p* = 0.037) but not of activity (F_1,27_ = 0.01, *p* = 0.918) and there was not a significant interaction effect (F_1,27_ = 1.87, *p* = 0.186). The contrasts revealed that the main effect of Time was largely driven by the changes in the rest state data (Rest: F_1,27_ = 7.86, *p* = 0.011; Active: F_1,27_ = 0.32, *p* = 0.578).

### 3.4. Experiment 4 (Motor Training)

The training only intervention showed a main effect of state for single-pulse MEPs (F_1,27_ = 4.52, *p* = 0.042) with no effect of time or interaction (*p*′s > 0.25). There was a main effect of time for the PMd-M1 paired-pulse data (F_1,27_ = 4.28, *p* = 0.048). There was no main effect of activity (F_1,27_ = 1.09, *p* = 0.307) nor was there an interaction (F_1,27_ = 0.29, *p* = 0.596). The contrasts revealed that neither of the activity states differed with Time, although Active trended towards significance (Rest: F_1,27_ = 1.18, *p* = 0.288; Active: F_1,27_ = 3.40, *p* = 0.076) (Figure 3).

## 4. Discussion

Beyond a widespread facilitation of MEPs, our data begin to reveal that cortical networks may be differentially modulated by the two V-PAS interventions. In general, resting V-PAS appears to modulate premotor to M1 projections which is congruent with previous findings [7]. Data in Figure 2 display some interesting observations to consider. The first is that SPOC to M1 projections tested at rest appear to be influenced by both resting and active V-PAS. This modulation is a significant effect following the active V-PAS condition but fails to reach significance following resting V-PAS likely due to baseline variability. Given that the effect size of either V-PAS intervention on resting ppTMS is nearly identical, it is likely there is a common effect present in both these measures which is independent of the type of V-PAS and depends rather only on the mechanisms of V-PAS itself as a causal factor. What this implies is SPOC to M1 projections are influenced by V-PAS regardless of whether the participant prepares reaching responses during the intervention. In this way, resting V-PAS will influence premotor cortex to M1 which will modulate visuomotor networks as known [7] but also target terminal projections from the SPOC to M1 network. This effect would likely be a result of premotor cortex to M1 projections between the two networks being physically shared or having spatially close projections; either of which option may expose both networks to effects from resting V-PAS. However, this does not explain the clearly selective influence on SPOC to M1 projections when participants are reaching during ppTMS following V-PAS + MT selectively. The primary difference to consider in this case is the presence of motor preparatory networks which will be accessed during the motor training V-PAS intervention. These networks encompass many regions within the brain, some of which include the supplementary motor area, premotor cortex, cingulate cortex, and parietal association regions [12] as well as basal ganglia [27] and cerebellum [28] to name a few. We contest the important difference is that these motor preparatory projections influence M1 in a way which exposes them to V-PAS effects selectively when they are recruited during the intervention (i.e., motor training V-PAS). Therefore, when motor preparation networks are engaged during ppTMS in the reaching condition, we see modulation upon MEP sizes following V-PAS without motor training. This modulation appears to be significantly altered following V-PAS with motor training however.

In all paired-pulse conditions between SPOC and M1, a significant increase in MEP size was observed compared to single pulse TMS alone (Figure 2). This finding suggests SPOC exhibits a net excitatory effect upon the primary motor cortex which is consistent with previous literature [21]. Interestingly, we found significant facilitatory effects of these projections during all conditions including rest whereas Vesia et al. [21] only reported facilitatory effects when a participant actively prepares a reaching movement. At rest, we observed MEPs on average 150% that of the test stimulus alone, and 250% when participants were actively preparing a reaching movement. This compares to previous research which observed no significant increase at rest, and 130% of the test stimulus during reach preparation [21]. Given the small sample size in this experiment, it is possible these results are due to intersubject variability. Alternatively, these variances may be accounted for by the differences between our tasks. Participants during our experiment were presented with a target on the screen in both the rest and active conditions. Furthermore, participants’ hands were always positioned in front of them, resting approximately 30 cm from the target. This contrasts with Vesia et al. [21], in which participants were positioned with the hand adjacent to the target during the resting condition. This subtle difference may have allowed our participants to subconsciously be planning the corresponding reach to the target even though they were instructed to consciously suppress that drive. In fact, previous research has postulated the existence of a visuomotor binding mechanism which operates without the need for an individual to overtly attend to the target [29]. It is suggested this model assists online control during tasks such as a reach to grasp using primarily visual reafference and proprioception distinct from other attentional binding processes. As this mechanism relies on comparison to an efference copy and is likely implicated during the visually guided reach task in our experiments, it is not unreasonable to expect activity within this network in preparation for a potential reach. In this way, it is possible SPOC to M1 projections within these motor-preparatory networks were activated throughout all conditions during our experiment which may account for the consistent facilitation effect.

We do not observe a significant effect of time within either session which suggests neither V-PAS intervention was altering cortical excitability in a way we could detect with single pulse MEPs. As we discuss below, there was variability in the individual responses to V-PAS such that some participants appeared to experience cortical facilitation and inhibition in others. Measures of SICI, ICF, and LICI were also hindered by the large variability across the sample. Consequently, we cannot conclude the presence or absence of any significant effects. A qualitative survey of the dataset points to a trend of disinhibition in SICI following V-PAS. We included these ppTMS measures to investigate the mechanism facilitating disinhibition of PMd to M1 projections. A future investigation would likely benefit by investigating these measures with a larger group including additional measures to investigate the potential factors (ie genetic polymorphisms) that influence responsiveness to V-PAS.

Regardless of best practices to recruit a homogenous sample, we observed a distinct difference between participants in their responses to the V-PAS intervention (Figure 3). Overall, half of the sample on average experienced a decrease in cortical excitability following the V-PAS interventions while the remaining half saw a relative increase in excitability from the intervention. Investigation into this trend did not yield any systematic cause attributed to study design or participant demographics. It is a possibility this variable response to V-PAS may represent a feature of the population we targeted. Future work to replicate these findings and isolate possible factors contributing to a participant’s response to V-PAS is indicated.

The net inhibitory influence exerted by the PMd over the primary motor cortex at rest state is a critical part of movement control and motor training [28,30]. Post intervention disinhibition of the PMd-M1 influence was expected [7,26] but a facilitation effect within the inhibitory connection (Figure 4) has not previously been reported to the best of our knowledge. Factors which could be attributed to the observed facilitation may be posited physiological changes resulting from the V-PAS paradigm, altering the interaction between the facilitatory neuronal populations of the PMd and the interneurons of M1. Our understanding of the motor preparatory and primary motor cortical neuronal populations is based on a model developed by Reis et al. [31]. This model shows the inhibitory and facilitatory neuronal populations in the PMd have primarily facilitatory neuron output which synapse onto interneurons (both inhibitory/facilitatory) in M1. It is these interneurons of M1 which synapse onto the corticospinal tract. The following conclusions postulated about the neuronal populations which experienced plasticity like changes, are based on this understanding.

Though studies have proven it possible to induce LTD in cortical areas associated with M1 [32] or mitigate GABAergic processes through movement training [33], the intervention used in this study aimed to induce LTP like plasticity and measure the glutamatergic plasticity processes, therefore cannot be used to draw any definitive conclusions about changes in GABAergic synapses or any potential LTD like plasticity.

With the motor training intervention alone (Experiment 4, Figure 3), adaptation only occurred in the active state. This was expected as active motor training has been shown to induce temporary LTP like changes in the premotor and motor cortices [34,35,36].

Since neither the excitability of M1, nor the expression of intracortical inhibition or facilitation was significantly altered by any of the interventions, we can postulate that the adaptation observed was not localized to the corticospinal neurons in M1, rather the projections connecting the SPOC/PMd and M1. Various motor training interventions have been shown to elicit excitability changes in motor preparatory areas and sensory regions which can be independent from [32] or precede the excitability changes seen in M1 [36]. This may explain why Suppa et al. [7] observed excitability changes in both PMd-M1 connections and in M1 after 600 trials of the VPAS intervention, while these experiments only observed excitability changes in the SPOC-M1 and PMd-M1 connections.

As expected, cortical excitability in the active state is elevated from rest state which is indicative of the motor preparation state in the motor preparatory areas and motor cortex. Adaptation in the active state after the V-PAS + motor training intervention was also expected; however, the characteristic variability of the pre-intervention active state paired-pulse data was too high to draw any significant conclusions about changes which occurred. A larger population group would be needed to verify a suspected significant facilitation.

Across all experiments, there was no compelling support for an additive effect in motor cortical excitability enhancement related to the combination of visual and motor preparatory afferent signalling during V-PAS, although the combination did produce a large enhancement of M1 excitability during motor preparation relative to V-PAS alone (Figure 2). As V-PAS relies on the precise timing of incoming cortical signalling with an external TMS pulse, we expected that bolstering the afference signalling would act to further strengthen the association between the now multiple cortical regions. The combination of V-PAS and motor training did produce more consistent enhancements of parietal and premotor influences on M1. Perhaps most surprisingly was the very large selective enhancement of SPOC-M1 facilitation during motor preparation to a visual target following V-PAS coupled with motor training mentioned above. Conversely, it is possible an “upper limit” exists in which once a certain threshold of afferent signalling is achieved, any additional afferents do not contribute to the resultant associative plasticity. Our experiments used a visual stimulus designed to maximally activate the visual cortex; a feature not commonly observed in the natural world. Studies which instead provide more naturally occurring stimuli such as vibration to a fingertip or more subtle visual cues, may find an additive effect across multiple sensory stimuli during V-PAS.

## Data Availability

The data presented in this study are available on request from the corresponding author.
